# Development of Potent Cellular and Humoral Immune Responses in Long-Term Hemodialysis Patients After 1273-mRNA SARS-CoV-2 Vaccination

**DOI:** 10.3389/fimmu.2022.845882

**Published:** 2022-03-23

**Authors:** Maria Gonzalez-Perez, Maria Montes-Casado, Patricia Conde, Isabel Cervera, Jana Baranda, Marcos J. Berges-Buxeda, Mayte Perez-Olmeda, Rodrigo Sanchez-Tarjuelo, Alberto Utrero-Rico, Daniel Lozano-Ojalvo, Denis Torre, Megan Schwarz, Ernesto Guccione, Carmen Camara, M Rosario Llópez-Carratalá, Emilio Gonzalez-Parra, Pilar Portoles, Alberto Ortiz, Jose Portoles, Jordi Ochando

**Affiliations:** ^1^ Centro Nacional de Microbiología, Instituto de Salud Carlos III, Madrid, Spain; ^2^ Department of Oncological Sciences, Icahn School of Medicine at Mount Sinai, New York, NY, United States; ^3^ Precision Immunology Institute, Icahn School of Medicine at Mount Sinai, New York, NY, United States; ^4^ Department of Immunology, Hospital La Paz, Madrid, Spain; ^5^ Department of Nephrology, Hospital Puerta de Hierro, Madrid, Spain; ^6^ Department of Nephrology, Instituto de Investigación Sanitaria (IIS)-Fundación Jimenez Díaz, Madrid, Spain; ^7^ Presidencia, Consejo Superior de Investigaciones Científicas (CSIC), Madrid, Spain

**Keywords:** COVID-19, SARS-CoV-2 vaccine, hemodialysis, chronic kidney disease, cellular response, humoral response

## Abstract

Long-term hemodialysis (HD) patients are considered vulnerable and at high-risk of developing severe acute respiratory syndrome coronavirus type 2 (SARS-CoV-2) infection due to their immunocompromised condition. Since COVID-19 associated mortality rates are higher in HD patients, vaccination is critical to protect them. The response towards vaccination against COVID-19 in HD patients is still uncertain and, in particular the cellular immune response is not fully understood. We monitored the humoral and cellular immune responses by analysis of the serological responses and Spike-specific cellular immunity in COVID-19-recovered and naïve HD patients in a longitudinal study shortly after vaccination to determine the protective effects of 1273-mRNA vaccination against SARS-CoV-2 in these high-risk patients. In naïve HD patients, the cellular immune response measured by IL-2 and IFN-ɣ secretion needed a second vaccine dose to significantly increase, with a similar pattern for the humoral response. In contrast, COVID-19 recovered HD patients developed a potent and rapid cellular and humoral immune response after the first vaccine dose. Interestingly, when comparing COVID-19 recovered healthy volunteers (HV), previously vaccinated with BNT162b2 vaccine to HD patients vaccinated with 1273-mRNA, these exhibited a more robust immune response that is maintained longitudinally. Our results indicate that HD patients develop strong cellular and humoral immune responses to 1273-mRNA vaccination and argue in favor of personalized immune monitoring studies in HD patients, especially if COVID-19 pre-exposed, to adapt COVID-19 vaccination protocols for this immunocompromised population.

## Introduction

Progression of chronic kidney disease (CKD) leads to the need of kidney replacement therapy such as hemodialysis (HD). Long-term HD patients are at higher risk of severe acute respiratory syndrome coronavirus-2 (SARS-CoV-2) infection associated with coronavirus 19 disease (COVID-19) (1–3). In addition, the overall mortality of SARS-CoV-2 increases from 3.2% in healthy individuals to >20% in HD patients (1, 4, 5). As HD prevalence is increasing worldwide (6), HD patients represent a public health problem and specific considerations should be given to immunization of HD patients against SARS-CoV-2 infection.

Recent humoral immunity studies on natural SARS-CoV-2 infection in HD patients reported that, while 75% seroconverted shortly after infection, >70% of these patients exhibit a rapid decline of IgG specific antibodies (7), indicating that HD patients develop short-term humoral immunity after COVID-19. On the contrary, cellular immunity data indicates that COVID-19 convalescent HD patients exhibit higher frequencies of SARS-CoV-2 reactive memory T cells (CD4^+^CD154^+^CD137^+^) that express IFN-γ and IL-2 in comparison to patients with normal kidney function, although this increase did not reach statistically significance in a single time-point observational case-control study (8).

Regarding vaccination in HD patients, previous studies demonstrated deficient immune responses against the Hepatitis B and Pneumococcus vaccines in high-risk groups (9–12). Interestingly, recent data reported two patients that did not respond to the Hepatitis B vaccine, did not mount antibody responses to the COVID-19 vaccine and developed severe COVID-19 infection after vaccination (13). This highlights the immunocompromised state of HD patients and the need for monitoring their specific humoral and cellular response against SARS-CoV-2 following vaccination. In this respect, some studies have reported (i) lower response rate to the vaccine, (ii) lower anti–spike antibody level and neutralizing capacity, and (iii) higher rate of COVID-19 infection compared to healthy volunteers (HV) after SARS-CoV-2 vaccination (14–16).

While the humoral immune response to COVID-19 mRNA vaccines in HD patients are currently under investigation, only a few studies have simultaneously studied the humoral and cellular immune responses in HD patients following vaccination. Bertrand and colleagues reported that 89% of HD patients developed anti-spike SARS-CoV-2 antibodies, while 100% displayed specific T cells response after full vaccination (17). More recent studies by Strengert and colleagues described significantly reduced IgG titers and IFN-γ release when compared to HV (18). These apparent contradictory studies may be due to differences in the study design, as data was obtained in a single time-point, arguing in favor of longitudinal experiments to fully understand the kinetics of the immune response of HD patients after SARS-CoV-2 vaccination.

Here, we investigated the effects of the 1273-mRNA SARS-CoV-2 vaccine on the humoral and cellular immune responses in a longitudinal study shortly after vaccination that included COVID-19 recovered and naïve HD patients and further compared the results with non-dialyzed healthy volunteers (HV).

## Methods

### Study Design

All 39 long-term hemodialysis (HD) patients were recruited at Hospital Puerta de Hierro and Fundación Jimenez Diaz (Madrid, Spain) between April and June, 2021. COVID-19 recovered patients were classified by RT-PCR and confirmed by their ability to react against membrane (M) peptide pools *in-vitro*. COVID-19-recovered patients (n=20) were 45% women and 55% men (mean age 65 years [SD 13.23]). Naïve patients (n=19) were 52.63% women and 47.36% male (mean age 64 years [SD 12.39]). All HD patients received the mRNA-1273 vaccine (Moderna). The study also includes 92 healthy volunteers (HV) that were fully vaccinated with BNT162b2 (Pfizer). The mean time since COVID-19 was 9.6 ± 3.1 months in HD patients. COVID-19-recovered healthy volunteers (HV) (n=45) were 76% women and 24% men (mean age 44.3 years [SD 16.90]). Naïve healthy volunteers (n=47) were 78% women and 22% men (mean age 39.9 years [SD 14.73]). The mean time since COVID-19 was 6.9 ± 4.1 months in HV. Blood samples were longitudinally collected at different time points: pre-vaccination (Pre), ten (d10) and twenty (d20) days after the first dose, followed by ten (d30) and twenty (d40) days after the second dose. HD patient characteristics are displayed in **Table 1**.

**Table 1:** Naïve and COVID-19 recovered HD patients on long-term hemodialysis present similar characteristics, except for diabetic nephropathy as a cause of end-stage renal disease and smoking.

Total	Naïve HD patients	N (partial)	COVID-19 HD patients	N (partial)
	N=19		N=20	
**Characteristics**				
Gender				
Male	47.36%	9	55%	11
Female	52.63%	10	45%	9
Age (Mean± SD)	64 ± 12.39		65.25 ± 13.23	
Active smoking (yes)	15.8%	3	0,15%	3
HD time, months (Mean± SD)	96.11 ± 102.48		81.41 ± 72.21	
Use of EPO	89.4%	17	100%	20
Previous kidney transplantation	26.31%	5	35,00%	7
				
**Comorbidities**				
Obesity	15.79%	3	30%	6
Hypertension	89.47%	17	95%	19
Diabetes mellitus	31.58%	6	45%	9
Ischemic heart disease	31.58%	6	15%	3
Dyslipidemia	68.42%	13	60%	12
**Cause of end-stage renal disease**				
Diabetic nephropathy	28.57%	2	40%	8
Hypertensive nephrosclerosis	5.26%	1	10%	2
IgA nephropathy	0		5%	1
Membranoproliferative glomerulonephritis	5.26%	1	10%	2

### Ethics Statement

Ethical approval of the study was obtained from the relevant authority - the Internal Review Board of Hospital Puerta de Hierro and Fundación Jimenez Diaz. Written informed consent was obtained from all participants prior to starting the study.

### SARS-CoV-2 Peptides

PepTivator ^®^ SARS-CoV-2 Peptide Pools (Miltenyi Biotec, Germany) of the Spike protein (S1, S+, and S) and the Membrane (M) protein were used to perform whole blood cultures.

### Whole Blood Cell Culture With SARS-CoV-2 Peptide Pools

Lithium heparinized blood samples were collected before the start of dialysis. On the same day, 320µl of whole blood were mixed with 80µl of RPMI and stimulated with PepTivator ^®^ SARS-CoV-2 Peptide Pools (S; 2µg/ml, M; 2µg/ml) or a DMSO control. After 16-20 hours of culture, supernatant (plasma) was collected and stored at -20°C for further cytokine quantification (19, 20).

### Cytokine Measurements and Analysis

Cytokine concentrations in the supernatants (plasma) were quantified using ELLA with microfluidic multiplex cartridges measuring IFN-ɣ and IL-2 release following the manufacturer’s instructions (ProteinSimple, San Jose, California). The cytokine levels present in plasma stimulated with DMSO were subtracted from the corresponding Peptide-pool stimulated samples as previously reported (20). Values higher than 32.7 pg/ml and 36.8 pg/ml were considered positive for IL-2 in naïve HV and HD patients, respectively. Values higher than 9.0 pg/ml and 27.6 pg/ml were considered positive for IFN-ɣ in naïve HV and HD patients **(** **Supplementary Figure 1** **).**

### Spike-Specific IgG Quantification

Liaison ^®^ SARS-CoV-2 TrimetricS IgG assay (DiaSorin, Stillwater, MN, USA) was used for semiquantitative detection of IgG directed against Spike glycoprotein in human plasma sample on the LIAISON XL (DiaSorin, Saluggia, Italy) with a measuring range from 4.81 BAU/ml to 2080.00 BAU/ml. As described by the manufacturer’s instructions, values over 2080.00 BAU/ml were diluted 1:20. Values over 33.8 BAU/ml were considered positive.

### ROC Curves

ROC curves and AUC values for predicting vaccination status based on IL-2 and IFNg levels were calculated using the ROCit (v2.1.1) R package in an R 4.0.3 environment (21). Predictions were generated by using the pre-vaccination time point as the control group and the twenty days post second dose (d40) time point as the case group. Optimal cutoffs were determined in ROCit using Youden’s index.

### Statistics

For IgG, IFN-ɣ and IL-2 determination, statistical comparison between groups was performed using ANOVA test in GraphPad Prism version 9.1.1 (GraphPad Software, La Jolla, CA) P (*≤0.05, ** ≤0.01, ***≤ 0.001, ****≤0.0001). Data are reported as mean ±SEM.

## Results

### Differential Cellular Immunity Effects of mRNA Vaccination in COVID-19 Recovered and Naïve HD Patients

**Figure 1:**
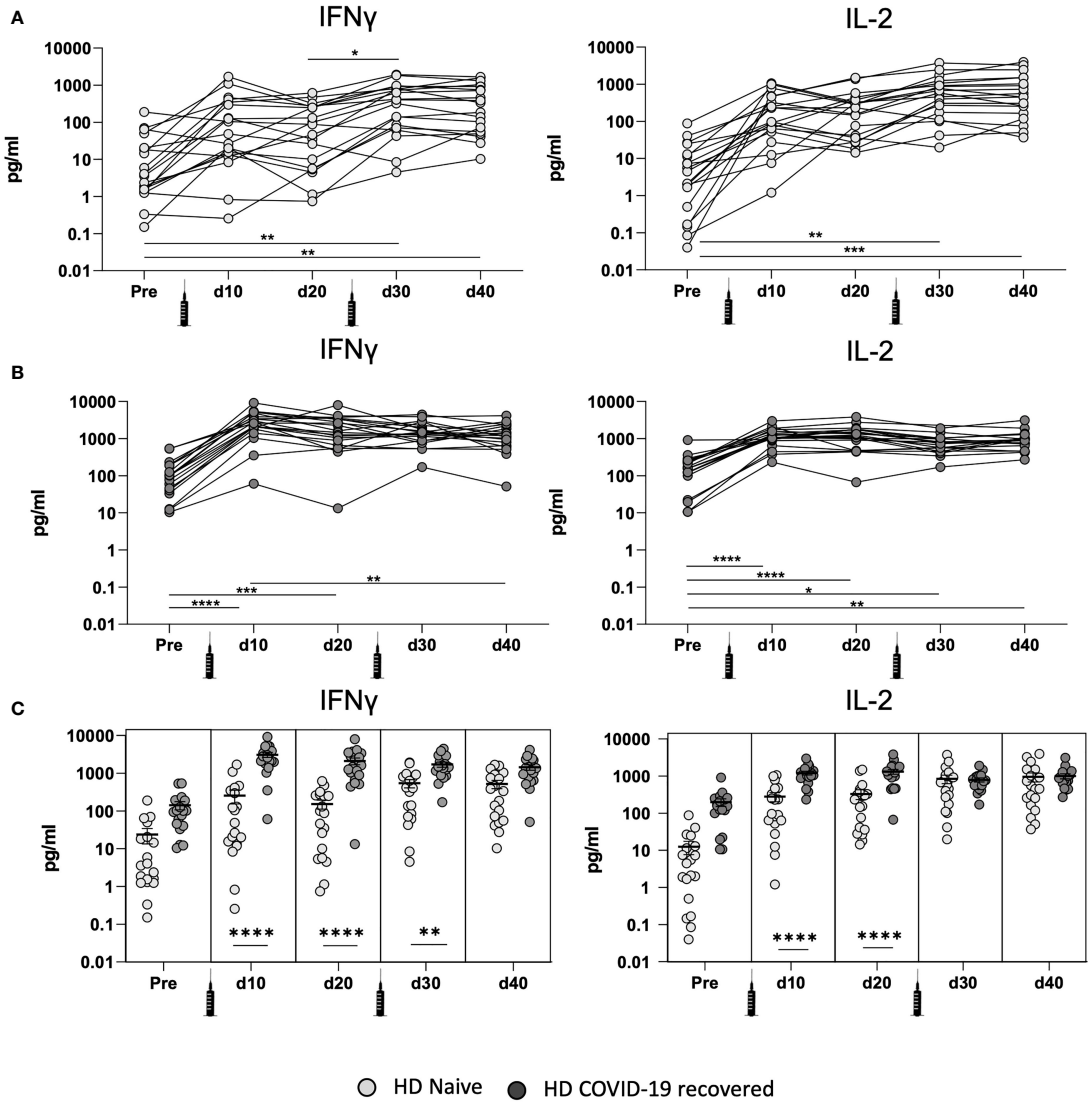
Development of cellular immunity after mRNA-1273 vaccination in COVID-19 recovered and naïve HD patients. **(A)** Quantification of IFN-ɣ and IL-2 production in the whole blood by naive HD patient cells at different time points: before vaccination (pre), after the first (d10 and d20) and second (d30 and d40) mRNA vaccine dose. **(B)** Quantification of IFN-ɣ and IL-2 production in the whole blood by COVID-19 recovered HD patient cells at different time points. **(C)** Comparison of IFN-ɣ and IL-2 production between naïve and COVID-19 recovered HD patients. All samples were analyzed after overnight stimulation of whole blood with SARS-CoV-2 peptide pools. IFN-ɣ and IL-2 levels were determined using ELLA single plex cartridges (n= 39; 19 naïve HD patients and 20 COVID-19 recovered HD patients). Values higher than 32.7 pg/ml and 36.8 pg/ml were considered positive for IL-2 in naïve HV and HD patients, respectively. Values higher than 9.0 pg/ml and 27.6 pg/ml were considered positive for IFN-ɣ in naïve HV and HD patients. * <0.05, ** <0.005, *** <0.0005, **** <0.0001.

In naïve HD patients without previous SARS-CoV-2 infection (**Figure 1A**) secretion of IFN-ɣ and IL-2 associated with cellular immunity was significantly increased after the second dose of the mRNA vaccine (d30). This suggests that IFN-ɣ and IL-2 secreting cells need the second vaccine dose to reach full immunity.

We next evaluated the cellular response in HD patients with previous SARS-CoV-2 infection (**Figure 1B**) and our results indicate that these patients achieved their peak of IFN-ɣ and IL-2 associated T cell responses ten days after the first vaccine dose (d10). Interestingly, the second dose did not significantly further increase the production of IFN-ɣ or IL-2, suggesting that only one dose may be necessary to achieve protection mediated by cellular immunity in COVID-19 recovered HD patients. These results suggest that HD patients with pre-existing immunity develop a more rapid and sustained cellular immune response against SARS-CoV-2 spike peptide pools after the first dose of the vaccine, consistent with our recent investigation in healthy volunteers (HV) (20).

When comparing the cellular immune response between HD patients with and without previous SARS-CoV-2 (**Figure 1C**) there are significant differences between IFN-ɣ and IL-2 after the first vaccination dose, but we did not observe differential cytokine secretion 20 days after the second vaccine dose (d40). Overall, the data suggests that HD patients develop potent cellular immunity in response to SARS-CoV-2 vaccination.

### Differential Humoral Immunity Effects of mRNA Vaccination in COVID-19 Recovered and Naïve HD Patients

**Figure 2:**
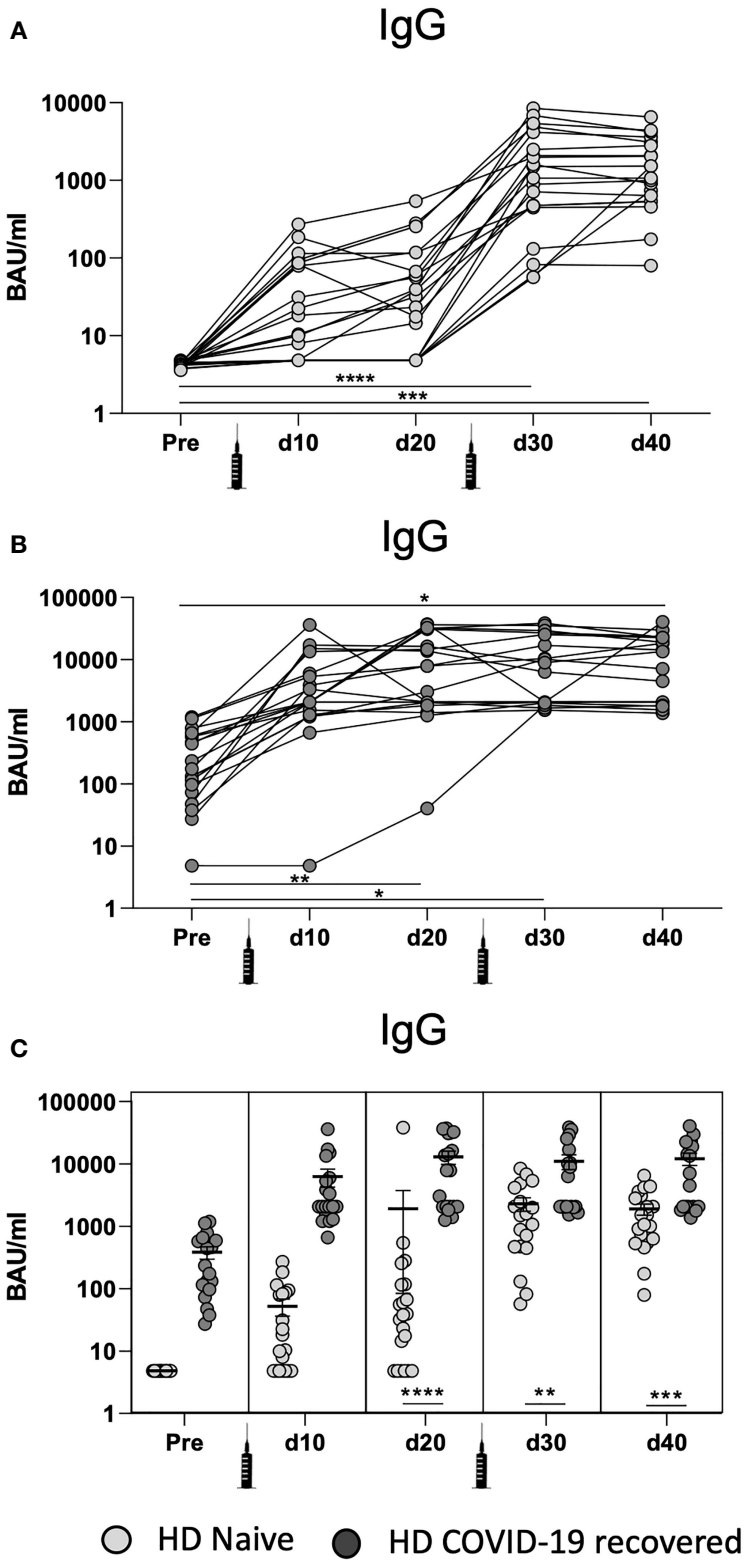
Development of humoral responses after mRNA-1273 vaccination in COVID-19 recovered and naïve HD patients. **(A)** Quantification of SARS-CoV-2 spike-specific IgG serum levels in naive HD patients at different time points: before vaccination (pre), after the first (d10 and d20) and second (d30 and d40) mRNA vaccine dose. **(B)** Quantification of SARS-CoV-2 spike-specific IgG serum levels in COVID-19 recovered HD patients at different time points. **(C)** Comparison of SARS-CoV-2 spike-specific IgG serum levels in naïve and COVID-19 recovered HD patients. Samples were measured with Liaison ^®^ SARS-CoV-2 TrimetricS IgG assay. Values higher than 33.8 BAU/ml were considered positive. * <0.05, ** <0.005, *** <0.0005, **** <0.0001.

In naive HD patients without previous SARS-CoV-2 infection (**Figure 2A**), the IgG-specific humoral immunity was significantly increased only after the second vaccine dose. This suggests that naïve HD patients may exhibit a similar cellular and humoral response patterns.

Next, we evaluated the humoral response in HD patients with previous SARS-CoV-2 infection (**Figure 2B**). These patients achieved their peak of IgG levels 20 days after the first vaccine dose (d20). In line with the cellular immune response, the second dose of the vaccine did not significantly increase the levels of IgG, suggesting that only one dose is necessary to achieve the peak humoral immunity in COVID-19 recovered patients.

When comparing the humoral immune response between HD patients with and without previous SARS-CoV-2 (**Figure 2C**), we observed that significant differences between IgG levels occur 20 days after the first vaccine dose (d20) and they are maintained longitudinally (d30 and d40). These results show that, while cellular immunity peaks 10 days after the first vaccine dose (d10) (**Figure 1B**), humoral IgG levels arise 20 days after the first vaccine dose (d20). Overall, the data indicate that HD patients develop potent humoral immunity in response to SARS-CoV-2 vaccination.

### Differential Correlation Between Humoral and Cellular Immunity in COVID-19 Recovered and Naïve HD Patients

**Figure 3:**
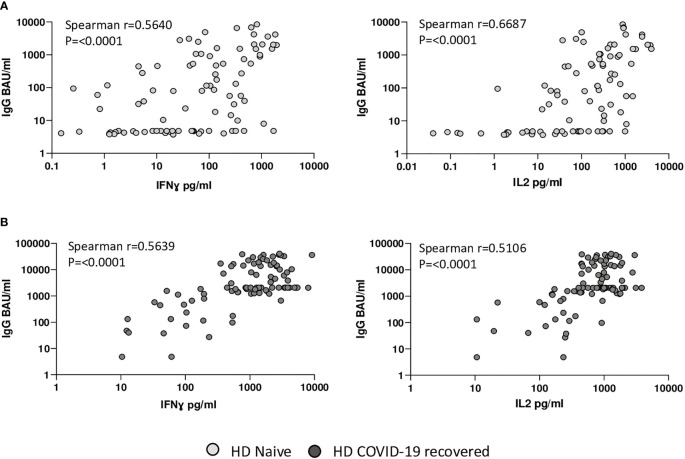
Relationship between cellular and humoral immune response in naïve and COVID-19 recovered HD patients. **(A)** Correlation between IFN-ɣ and IL-2 levels and SARS-CoV-2 spike-specific IgG serum levels in naïve HD patients. **(B)** Correlation between IFN-ɣ and IL-2 levels and of SARS-CoV-2 spike-specific IgG serum levels in COVID-19 recovered HD patients. Figure shows all data points available from any time point for all patients.

In naïve HD patients, we observed a significant correlation between the overall humoral (IgG) and the cellular (IFN-ɣ and IL2) immune responses (**Figure 3A**). Our results are comparable to other studies examining vaccination responses to BNT162b2, which observed correlation between T-cell and B-cell responses in naïve HD patients (Spearman’s rho=0·56) (18). HD COVID-19 recovered HD patients also displayed a significant correlation between the humoral (IgG) and the cellular (IFN-ɣ and IL2) immune responses (**Figure 3B**). In conclusion, **Figure 3** indicates a strong correlation between the cellular and humoral immunity in both COVID-19 recovered and naïve HD patients.

### Humoral and Cellular Immunity in HD Patients and HV Individuals

**Figure 4:**
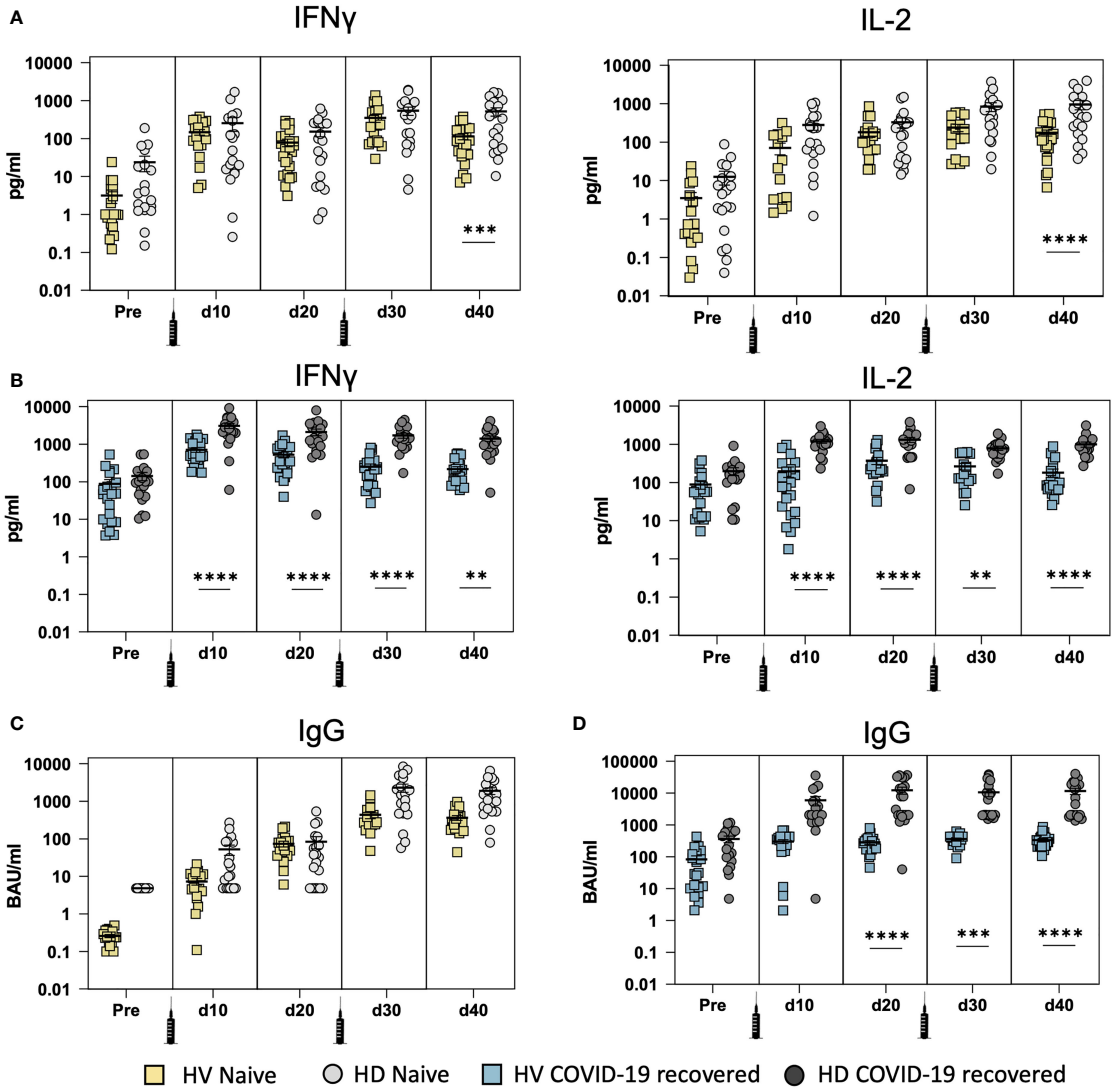
Development of cellular and humoral immunity after SARS-CoV-2 vaccination in COVID-19 recovered and naïve hemodialysis (HD) patients vaccinated with mRNA-1273 vaccine (100 µg/dose) and in healthy volunteers (HV) vaccinated with BNT162b2 (30 µg/dose). **(A)** Comparison of IFN-ɣ and IL-2 production between naïve HD patients and HV at different time points: before vaccination (pre), after the first (d10 and d20) and second (d30 and d40) mRNA vaccine dose. **(B)** Comparison of IFN-ɣ and IL-2 production in COVID-19 recovered HD patients and HV at different time points. **(C)** Comparison of SARS-CoV-2 spike-specific IgG serum levels in naïve HD patients and HV. **(D)** Comparison of SARS-CoV-2 spike-specific IgG serum levels in COVID-19 recovered HD patients and HV. ** <0.005, *** <0.0005, **** <0.0001.

We next compared the humoral and cellular immune response between mRNA-1273 vaccinated HD patients (100 µg/dose) and BNT162b2 vaccinated healthy volunteers (HV) (30 µg/dose) with and without previous SARS-CoV-2 infection. Our results demonstrate that naïve HD patients without previous SARS-CoV-2 infection (**Figure 4A**) exhibit a significant increase in IFN-ɣ and IL-2 production 20 days after the second vaccine dose (d40), in comparison with HV. We further compared the cellular response in COVID-19 recovered patients with previous SARS-CoV-2 infection (**Figure 4B**) and our results indicate a significant increase in IFN-ɣ and IL-2 production after the first vaccine dose (d10), which is maintained longitudinally. Overall, these results indicate that naïve HD patients exhibit a significant increase in the cellular immune response in comparison with HV. However, this observation is likely be associated with the mRNA vaccine dosage on the magnitude of the induced cellular immune response.

Finally, we compared spike-specific IgG levels in both vaccinated HD patients and HV individuals with and without previous SARS-CoV-2 infection. Our results demonstrate that naïve HD patients without previous SARS-CoV-2 infection (**Figure 4C**) exhibit similar IgG levels to HV and no significant differences between HD and HV were observed. On the contrary, HD patients with previous SARS-CoV-2 infection (**Figure 4D**) display a significant increase in spike-specific IgG production after the first dose of the vaccine (d20) that is maintained longitudinally. This confirms that COVID-19 recovered HD patients develop strong humoral immunity in response to SARS-CoV-2 vaccination.

## Discussion

In this study, we investigated the effects of the mRNA-1273 vaccine on the SARS-CoV-2 specific cellular and humoral immune responses in HD patients with and without previous SARS-CoV-2 infection in a longitudinal study shortly after vaccination and compared the results to a HV cohort. Our results indicate that naïve HD patients without previous SARS-CoV-2 infection develop an effective cellular and humoral immune response after the second dose of the vaccine, while HD patients with previous SARS-CoV-2 infection exhibit a potent and rapid immune response after the first dose. Interestingly, HD patients display an overall significant increase in the production of IFN-ɣ, IL-2 and IgG in comparison to HV.

Previous studies evaluating the humoral response in naïve HD patients reported a favorable but profoundly lower SARS-CoV-2 spike protein antibody response in comparison with a non-dialysis cohort (i.e. median 253 versus 1756 U/mL, P < 0.001) (22–24). Consistent with these results, Simon et al. described that, while 80% of HD patients developed a humoral immunity (>29 U/ml), these patients had significantly lower anti-SARS-CoV-2 S antibody titers than control patients 21 days after vaccination (median was 171 U/mL for dialysis patients and 2500 U/mL for controls) (16). Similar frequencies of seroconversion were observed by others, in which 20-30% of patients on dialysis had a suboptimal humoral response to vaccination or were non-responders (14, 25, 26). On the contrary, other studies have described strong humoral immunity in response to complete vaccination in naïve HD patients reporting a remarkably high seroconversion rate of ≥95% (27–29), although a direct comparison between the IgG values in HD *vs*. HV was not reported. One possible explanation for the different conclusions in the above studies may be the limited number of HD patients enrolled in some of the studies. Our results are in line with studies suggesting high seroconversion rates in naïve HD patients, but further demonstrate similar IgG levels when compared to HV.

HD patients with previous SARS-CoV-2 infection develop robust humoral responses and earlier studies have reported similar seroconversion rates and IgG levels between HD patients and healthy volunteers with previous infection (HD: 51475 U/mL; HV: 10650 U/mL, P = 0.024) (22). In addition, others have reported that COVID-19 recovered HD patients reach their IgG peak levels after the first dose in comparison to naïve HD patients (29). These observations are consistent with our results, which demonstrate that COVID-19 recovered HD patients exhibit strong and fast humoral immunity after the first vaccine dose. However, we observed a significantly higher humoral response in HD patients when compared with HV, which has not been previously reported and argues in favor of additional studies that distinguish between naïve and SARS-CoV-2 infected HD patients.

With regards to cellular immunity, some studies reported a lower IFN-ɣ production three weeks after the second vaccine dose in naïve HD patients compared to HV, as only 71% of HD patients responded to SARS-CoV-2-specific *in vitro* T cell activation by interferon-ɣ release assay (IGRA) (18). Comparable frequencies of decreased IFN-ɣ production were observed by other authors. Schrezenmeier and colleagues reported that 67% of naïve HD patients displayed significantly lower levels of IFN-ɣ release than healthy controls (93%) (24), while similar findings were observed using flow cytometry by Broseta and colleagues, in which activated CD4^+^ T cells expressing intracellular IFN-ɣ were observed only in 62% of naïve HD patients (28). Other studies have noted no difference between healthy controls and HD patients with regards to cellular immune response (30). On the contrary, recent studies have described that naïve HD patients exhibit an adequate T cell immunity five weeks after the second vaccine dose as assessed by IGRA and flow cytometry (27). Consistent with these results, Bertrand and colleagues described that all naïve HD patients develop T cell immune response in after the second vaccine dose measured by ELISpot (17). Our results are consistent with the later studies which report high percentages of T cell immunity after vaccination, but we further extend those findings and provide qualitative IFN-ɣ and IL-2 production measurements, which indicate for the first time that HD patients produce significantly higher pro-inflammatory cytokines than HV. We did not find prior studies that compared the cellular immunity in response to SARS-CoV-2 vaccination between COVID-19 recovered and HD patients without previous infection with SARS-CoV-2.

Taken together, we conclude that HD patients mount strong cellular and humoral immune responses after mRNA-1273 SARS-CoV-2 vaccination despite their immunocompromised condition. Unexpectedly, longitudinal immune monitoring of HD COVID-19 recovered patients revealed a potentially excessive cellular immune response that may be associated with a pro-inflammatory syndrome observed in HD patients in comparison to HV. While naïve HD patients may benefit from a third vaccine dose as described by Bensouna et al (31), COVID-19 recovered HD may be at risk of developing T cell exhaustion arguing in favor of personalized immune monitoring studies in HD patients. In Bensouna’s study, HD patients with a history of symptomatic COVID-19 were excluded and the third vaccine dose appeared to have a diminished benefit in patients who had already developed good humoral responses after two vaccine doses. Interestingly, in 4 patients that were positive for anti-nucleocapsid antibodies, the levels of anti-spike humoral response decreased after the third vaccine dose (anti-spike after the 2nd dose, 165,565 AU/ml; anti-spike after the 3rd dose, 116,110 AU/ml), which suggests that HD patients with previous SARS-CoV-2 infection may be spared from additional booster vaccine doses. Other studies have described an enhanced humoral response after the third dose in HD patients independently of previous SARS-CoV-2 infection but specifically in those with lower antibody titers after the second dose (32). Our study provides a broader assessment of the efficacy and dynamics of SARS-CoV-2 vaccination in HD patients, providing evidence that boost vaccination may not be necessary for HD patients with a history of previous SARS-CoV-2 infection.

As a limitation to our study, our HD patients were vaccinated with mRNA-1273 (Moderna) while HV individuals were vaccinated with BNT162b2 (Pfizer). Some studies have indicated that dialysis patients vaccinated with BNT162b2 had higher prevalence of no detectable or diminished IgG response, compared with patients vaccinated with mRNA1273 (33). In addition, Kaiser and colleagues described that patients vaccinated with mRNA-1273 display a 3-fold significantly higher spike-specific IgG titers (34). Furthermore, a lower seroconversion rate has been described in naïve HD patients vaccinated with BNT16b2 vaccine (88%) compared to mRNA-1273 vaccine (97%) (27). However, the absolute indicators of cellular and humoral immunity in HD and HV of our study are comparable, as we used the same methodological approaches to obtain the data.

To our knowledge, this is the first longitudinal study investigating the differential effects of cellular and humoral immunity in response to mRNA vaccination, distinguishing between HD patients’ previous history of SARS-CoV-2 infection and comparing the results with a HV cohort. Our work aims at providing additional scientific evidence and understanding of the immune response to SARS-CoV-2 infection and vaccination to further reduce the hesitancy of COVID-19 vaccination in HD patients (35).

## Data Availability Statement

The original contributions presented in the study are included in the article/**Supplementary Material**, further inquiries can be directed to the corresponding authors.

## Ethics Statement

Ethical approval of the study was obtained from the relevant authority - the Internal Review Board of Hospital Puerta de Hierro and Fundación Jimenez Diaz. Written informed consent was obtained from all participants prior to starting the study. The patients/participants provided their written informed consent to participate in this study.

## Author Contributions

MG-P, MM-C, PC, IC, JB, and MB-B performed cellular assays. MP-O performed humoral assays. RS-T and DL-O, DT and MS organized the database and performed the statistical analysis. EG, CC, ML-C, EG-P, PP, AO, JP, and JO contributed to conception and design of the study. All authors contributed to the article and approved the submitted version.

## Funding

Funding was obtained from Instituto de Salud Carlos III (ISCIII) RICORS program to RICORS2040 (RD21/0005/0001), FEDER funds; Acción Estratégica en Salud Intramural (AESI), Instituto de Salud Carlos III, grant number AESI PI21CIII_00022 to PP and Healthstar-plus -REACT-UE Grant through Segovia Arana Research Institute Puerta de Hierro Majadahonda-IDIPHIM. JO is a member of VACCELERATE (European Corona Vaccine Trial Accelerator Platform) Network, which aims to facilitate and accelerate the design and implementation of COVID-19 phase 2 and 3 vaccine trials. JO is a member of the INsTRuCT under the MSC grant agreement Nº860003 (Innovative Training in Myeloid Regulatory Cell Therapy) Consortium, a network of European scientists from academia and industry focused on developing innovative immunotherapies.

## Conflict of Interest

The authors declare that the research was conducted in the absence of any commercial or financial relationships that could be construed as a potential conflict of interest.

## Publisher’s Note

All claims expressed in this article are solely those of the authors and do not necessarily represent those of their affiliated organizations, or those of the publisher, the editors and the reviewers. Any product that may be evaluated in this article, or claim that may be made by its manufacturer, is not guaranteed or endorsed by the publisher.

## Supplementary Material

Supplementary Figure 1 Optimal cutoff values for IFN-ɣ and IL-2. **(A)** Receiver operating characteristic (ROC) curves for predicting vaccination status from IFN-ɣ and IL-2 levels in HV and HD patients with and without prior COVID-19 infection. Area under curve (AUC) and optimal cutoff values are displayed. **(B)** Bar plot with optimal cutoffs for predicting vaccination status from IFN-ɣ and IL-2 levels in HV and HD patients with and without prior COVID-19 infection. **(C)** Histogram displaying individual values of IFN-ɣ and IL2 in HV and HD patients with and without prior COVID-19 infection before vaccination (pre) and twenty days after the second vaccination dose (post). Vertical lines indicate the optimal cutoff values.

## Abbreviations

HD, Hemodialysis; HV, Healthy Volunteers; COVID-19, Coronavirus disease 2019; IFN-ɣ, Interferon gamma.
