# Supplementary figures and images for: Development of Potent Cellular and Humoral Immune Responses in Long-Term Hemodialysis Patients After 1273-mRNA SARS-CoV-2 Vaccination
Source: Front Immunol. 2022 Mar 23;13:845882. doi: 10.3389/fimmu.2022.845882 (PMC8983822; doi:10.3389/fimmu.2022.845882)

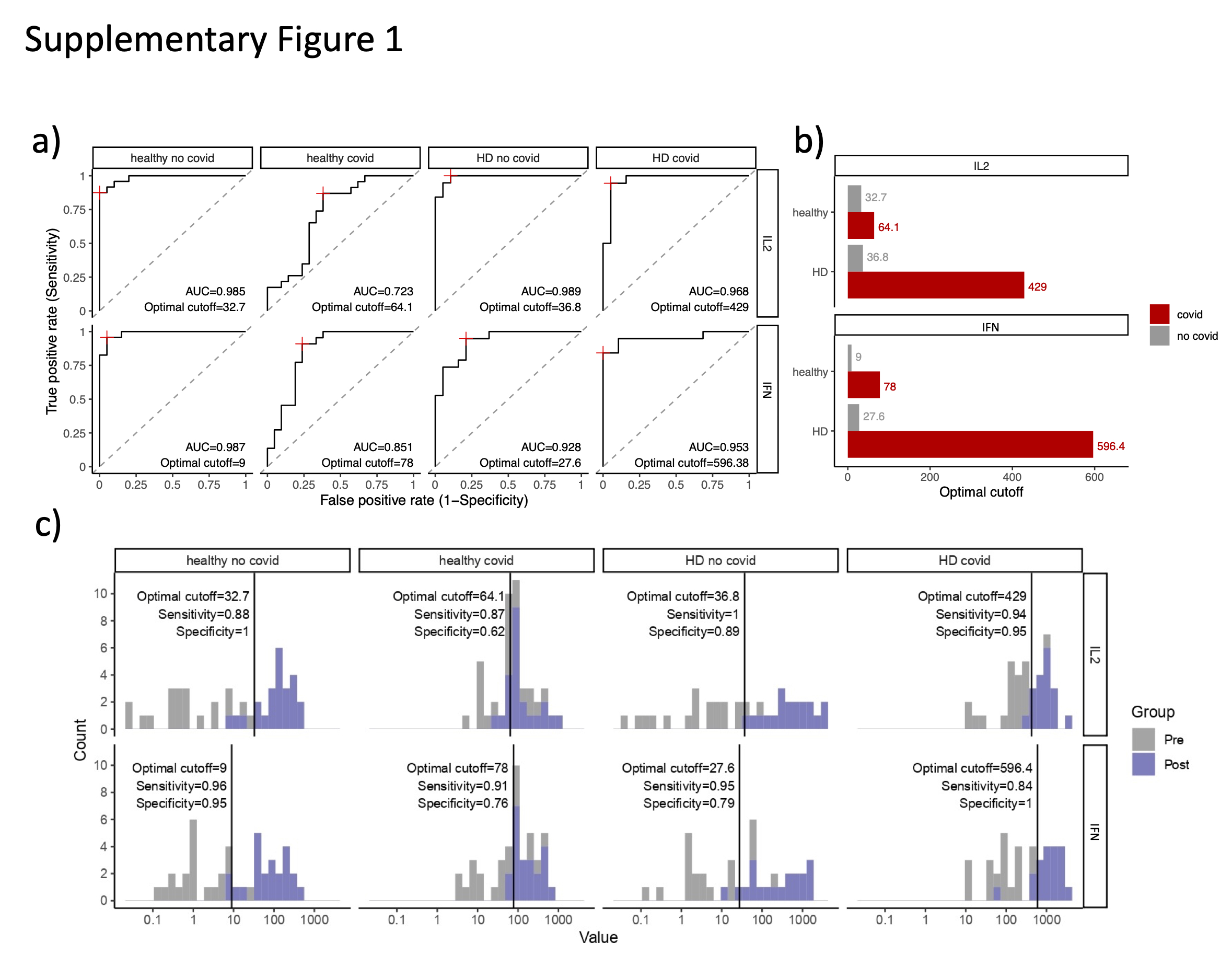

Supplement: Supplementary Figure 1 — Optimal cutoff values for IFN-ɣ and IL-2. (A) Receiver operating characteristic (ROC) curves for predicting vaccination status from IFN-ɣ and IL-2 levels in HV and HD patients with and without prior COVID-19 infection. Area under curve (AUC) and optimal cutoff values are displayed. (B) Bar plot with optimal cutoffs for predicting vaccination status from IFN-ɣ and IL-2 levels in HV and HD patients with and without prior COVID-19 infection. (C) Histogram displaying individual values of IFN-ɣ and IL2 in HV and HD patients with and without prior COVID-19 infection before vaccination (pre) and twenty days after the second vaccination dose (post). Vertical lines indicate the optimal cutoff values. [file Image_1.jpeg]
